# Spectroscopic and Docking Studies on the Binding of Liquiritigenin with Hyaluronidase for Antiallergic Mechanism

**DOI:** 10.1155/2016/9178097

**Published:** 2016-05-26

**Authors:** Hua-jin Zeng, Ran Yang, Jing You, Ling-bo Qu, Yan-jun Sun

**Affiliations:** ^1^School of Pharmaceutical Sciences, Zhengzhou University, Zhengzhou 450001, China; ^2^College of Chemistry and Molecular Engineering, Zhengzhou University, Zhengzhou 450001, China

## Abstract

The inhibitory effect of liquiritigenin on hyaluronidase and its binding mechanism were investigated systematically by UV-vis absorption, fluorescence, and molecular modeling approaches. These results indicated that liquiritigenin could interact with hyaluronidase to form a liquiritigenin-hyaluronidase complex. The binding constant, number of binding sites, and thermodynamic parameters were calculated, which indicated that liquiritigenin could spontaneously bind with hyaluronidase mainly through electrostatic and hydrophobic interactions with one binding site. Synchronous fluorescence, three-dimensional fluorescence, and molecular docking results revealed that liquiritigenin bound directly to the enzyme cavity site and this binding influenced the microenvironment of the hyaluronidase activity site, resulting in reduced hyaluronidase activity. The present study provides useful information for clinical applications of liquiritigenin as a hyaluronidase inhibitor.

## 1. Introduction

Hyaluronidase (HAase), an enzyme that depolymerizes the polysaccharide hyaluronic acid in the extracellular matrix of connective tissue, is found both in organs and in body fluids [1]. The enzyme is known to be involved in allergic reactions [2], cancer metastasis [3], inflammation, and petechial hemorrhages following its injection in mesentery preparations and also an increase in the permeability of the vascular system [4]. This information seemed to indicate that potent HAase inhibitory substances might have antiallergic, anti-inflammatory, and anticancer effects and could become leading compounds in the development of new antiallergic drugs. Therefore, on the basis of this information, some researchers have been devoted to searching and detecting the inhibitory effects of some natural products against HAase [5–7].

**Figure 1:**
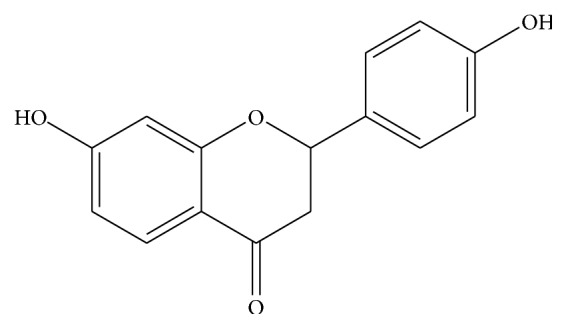
Molecular structure of liquiritigenin.

Due to its invigorating spleen, replenishing *qi* (a term of traditional Chinese medicine), clearing heat, and removing toxic substance, liquorice root (*Glycyrrhiza glabra* L.) has been used as a traditional Chinese medicine (TCM) for treatment of many diseases, such as arresting coughing, reducing fever, alleviating urgency, comforting the stomach, and potentiating the effects of various other herbs about 2000 years [8]. Liquiritigenin (structure shown in Figure 1), a major active component of liquorice root, belongs to flavonoid and is used clinically for the treatment of allergic disorder and inflammation [9]. These results indicated that liquiritigenin might also possess potential ability to inhibit the activity of HAase. Some previous studies have proved that several flavonoids, such as apigenin, luteolin, kaempferol, quercetin, and rutin [10–12], had the inhibitory effect on HAase and their inhibitory ability was closely related to their structure. However, to our knowledge, these studies were limited to the enzymatic activity assay and the inhibitory mechanism of liquiritigenin on HAase has not been investigated.

In this study, the inhibitory effect and interaction mechanism of liquiritigenin with HAase were investigated by UV-vis absorption, fluorescence, and molecular modeling approaches. The binding constant, thermodynamic parameters, the special binding site, and the effect of liquiritigenin on HAase conformation were evaluated. The aim of this work was to determine the inhibitory mechanism of liquiritigenin on HAase activity and to provide useful information for clinical applications of liquiritigenin as a HAase inhibitor.

## 2. Experimental

### 2.1. Reagents

HAase was purchased from Sigma-Aldrich Chemical Co. (USA) and its stock solution (1.0 × 10^−5^ mol L^−1^) was made in sodium phosphate buffer (0.2 mol L^−1^, pH = 7.4) and then diluted to the required concentrations with the buffer. Liquiritigenin was obtained from the National Institute for the Control of Pharmaceutical and Biological Products (Beijing, China), and its stock solution (1.0 × 10^−3 ^mol L^−1^) was prepared by dissolving its crystals in methanol and constant-volume with the sodium phosphate buffer. All reagents and solvents were of analytical reagent grade, and ultrapure water was used throughout the experiment. All stock solutions were stored at 4°C.

### 2.2. Apparatus

The UV-vis absorption measurements were performed with a Shimadzu UV-2450 spectrophotometer equipped with 1.0 cm quartz cells. Fluorescence spectra were recorded on a Hitachi spectrofluorometer Model F-2500 equipped with 1.0 cm quartz cells. All experiments, unless otherwise specified, were carried out at room temperature.

### 2.3. Procedures

#### 2.3.1. HAase Activity Assay

Effect on HAase enzyme activity was measured by the modified Morgan-Elson method with some modifications [12, 13]. Briefly, HAase enzyme (500 U mL^−1^) was incubated with the test samples for 20 min at 37°C. Then, 12.5 mM calcium chloride was added to the mixture and incubated for 20 min at 37°C again. The hyaluronic acid sodium solution (0.5 mg mL^−1^) was added and incubation at 37°C for 40 min. After the addition of sodium hydroxide and sodium borate, the reaction mixture was heated in a boiling water bath for 5 min to stop the enzyme reaction. After cooling to room temperature,* p*-dimethylaminobenzaldehyde (DMAB) reagent was added and incubated at 37°C for 30 min when colour developed. The absorbance at 530 nm of the clear supernatant was measured. Test samples were replaced by the buffer solution for the control. The HAase inhibition rate was calculated using the following formula:

(1) $$\begin{array}{c} \text{Inhibition }\left(\;\%\;\right)=\frac{\left({OD}_{control}-{OD}_{sample}\right)}{{OD}_{control}}\times 100. \end{array}$$

#### 2.3.2. Fluorescence Measurement

The fluorescence measurements were carried out by successive addition of the solution of liquiritigenin to a fixed amount of HAase (to give a final concentration of 2.0 × 10^−6^ mol L^−1^) in each tube. The final volume was made up to 5.0 mL with sodium phosphate buffer. Thus, a series of solutions containing different amount of liquiritigenin and a definite amount of HAase were obtained. The fluorescence spectra were then measured (excitation at 280 nm and emission wavelengths of 290–450 nm) at 293 and 310 K, respectively. All solutions were mixed thoroughly and kept for 20 min before measurement.

In addition, the fluorescence intensity will be reduced if the compounds added in fluorescence determination system have ultraviolet absorption at excitation and emission wavelength. Thus, in this work, all fluorescence intensities were corrected for inner filter effect by using the following equation [14]:

(2) $$\begin{array}{c} F_{\text{cor}}=F_{\text{obs}}\times 10^{\left(A_{ex}+A_{em}\right)/2}, \end{array}$$

where *F*_cor_ is the fluorescence intensity corrected, *F*_obs_ is the fluorescence intensity calculated in experiment, and *A*_ex_ and *A*_em_ are the absorbance of silybin in fluorescence determination system at excitation wavelength (280 nm) and the emission wavelength, respectively.

The synchronous fluorescence spectra of HAase in the presence of liquiritigenin were recorded at 293 K and the *D*-value (Δ*λ*) between excitation wavelength and emission wavelength was stabilized at 15 or 60 nm. The three-dimensional fluorescence spectra were performed under the following conditions: the emission wavelength range was selected from 270 to 500 nm, the initial excitation wavelength was set to 200 nm, and the scanning number was 15 with the increment of 10 nm.

#### 2.3.3. Molecular Docking Investigation

The docking program AutoDock 4.0 was used to explore the probable interaction between liquiritigenin and HAase. The 3D structure of liquiritigenin was generated in Chem3D Ultra 8.0, and the crystal structure of HAase (PDB ID: 2PE4) was retrieved from the RCSB Protein Data Bank (http://www.rcsb.org/pdb/home/home.do). To carry out docking simulations, a grid box was defined to enclose the active site with dimensions of 126 Å × 126 Å × 126 Å and a grid spacing of 0.375 Å. The grid maps for energy scoring were calculated using AutoGrid. Docking calculations were performed using the Lamarckian genetic algorithm (LGA) and the search parameters were set to 100 times. From the docking results, the best scoring docked model (the lowest energy conformation) of a compound was chosen to represent its most favorable binding mode predicted by AutoDock.

## 3. Results and Discussion

### 3.1. Effect of Liquiritigenin on HAase Activity

HAase plays an important role in allergic diseases and inhibition on the HAase would be an effective antiallergic therapy. In order to evaluate the antiallergic activities of liquiritigenin, the inhibitory effect of it on HAase was investigated. With the increase of liquiritigenin concentration, the relative HAase activities were decreased significantly and the 50% relative activity (IC_50_) was estimated to be 680 ± 43 *μ*mol L^−1^. The result implies that liquiritigenin can inhibit HAase activity.

### 3.2. Characterization of the Binding Interaction of Liquiritigenin with HAase by Fluorescence Measurements

**Figure 2:**
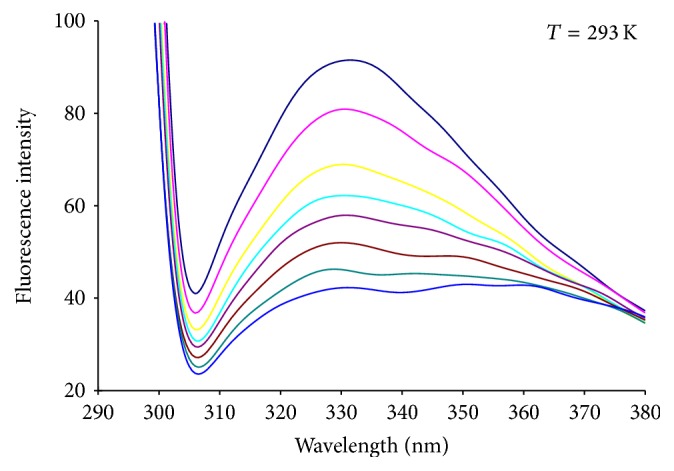
The fluorescence emission spectra of HAase in the presence of increasing amounts of liquiritigenin. Peak from up to down *C*_liquiritigenin_ = (0, 4.0, 8.0, 12.0, 16.0,20.0, 24.0, 30.0) × 10^−6^ mol L^−1^, *C*_HAase_ = 2.0 × 10^−6^ mol L^−1^.

The significant inhibitory activity of liquiritigenin on HAase suggests that liquiritigenin may directly bind to the enzyme. Thus, fluorescence measurements were used to further determine the interaction between liquiritigenin and HAase.  Figure 2 shows the fluorescence quenching spectra of HAase induced by different concentrations of liquiritigenin with the excitation wavelength of 280 nm. It can be seen from Figure 2 that liquiritigenin in phosphate buffer emitted little fluorescence under the experimental conditions, while HAase had a strong fluorescence emission peaking at 338 nm after being excited with a wavelength of 280 nm. When a fixed concentration of HAase was titrated with different concentration of liquiritigenin, a remarkable intrinsic fluorescence decrease of HAase was observed. The results indicate that liquiritigenin may interact with HAase and quench its intrinsic fluorescence.

**Figure 3:**
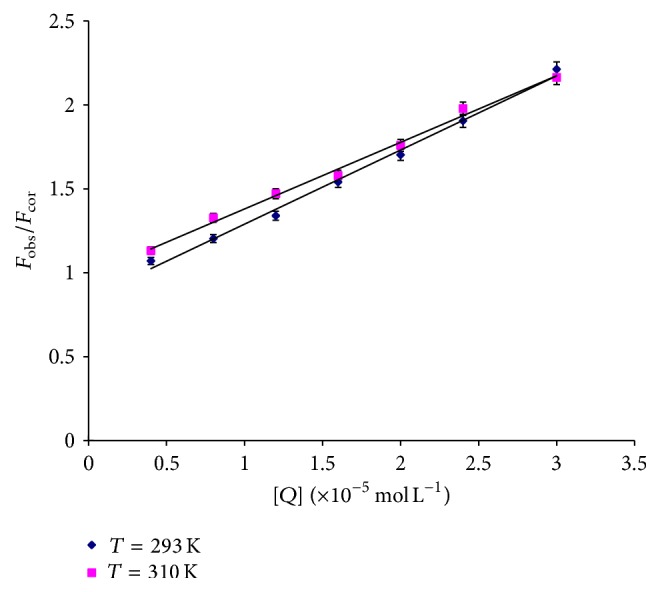
Stern-Volmer plots for the quenching of HAase by liquiritigenin at different temperatures (*n* = 3).

To elucidate further the quenching mechanism of HAase induced by liquiritigenin, the fluorescence quenching data were analyzed on the basis of the Stern-Volmer equation [15] as follows:

(3) $$\begin{array}{c} \frac{F_{\text{obs}}}{F_{\text{cor}}}=1+K_{q}\tau_{0}\left[\;Q\;\right]=1+K_{\text{sv}}\left[\;Q\;\right], \end{array}$$

where *K*_sv_, *K*_*q*_, *τ*_0_, and [*Q*] are the Stern-Volmer dynamic quenching constant, the quenching rate constant of the biomolecule, the average lifetime of the fluorophore in the absence of quencher (*τ*_0_ = 10^−8^ s) [16], and the concentration of quencher, respectively. Hence, (3) was applied to determine *K*_sv_ and *K*_*q*_ by linear regression of *F*_obs_/*F*_cor_ − 1 versus [*Q*].  Figure 3 shows the Stern-Volmer plots for the HAase fluorescence quenching by the liquiritigenin and the calculated *K*_sv_ and *K*_*q*_ values are summarized in Table 1. As shown in Table 1, the *K*_sv_ values inversely correlate with temperature, indicating that the probable quenching mechanism between HAase and liquiritigenin is not initiated by dynamic collision but by complex formation. Moreover, the corresponding *K*_*q*_ values at 293 and 310 K were all higher than the limiting diffusion constant of the bimolecule (2.0 × 10^10^ L mol^−1^ s^−1^), which confirmed that the fluorescence quenching was not the result of dynamic collision quenching, rather a consequence of static quenching.

**Figure 4:**
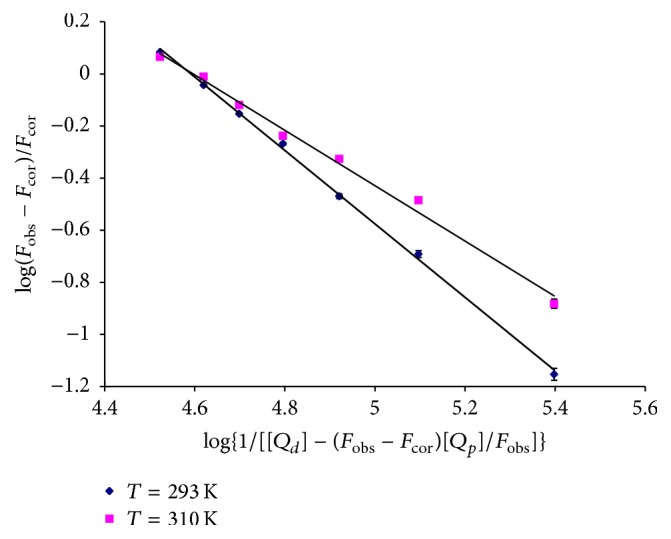
Double-log plots of liquiritigenin quenching effect on HAase fluorescence at different temperatures (*n* = 3).

In a static quenching process, when small molecules bind independently to a set of equivalent sites on a macromolecule, the equilibrium between free and bound molecules was given by the following equation [17, 18]:

(4) log⁡Fobs−FcorFcor=nlog⁡Ka−nlog⁡1Qd−Fobs−FcorQp/Fobs,

where *K*_*a*_ is static quenching constant, *n* is the number of binding sites per HAase molecule, and [*Q*_*d*_] and [*Q*_*p*_] are the concentration of drug molecule (liquiritigenin) and protein (HAase), respectively. The values of binding constant and the number of binding sites under different temperatures can be obtained from the intercept and slope of plots of log⁡(*F*_obs_ − *F*_cor_)/*F*_cor_ versus log⁡{1/[[*Q*_*d*_]−(*F*_obs_ − *F*_cor_)[*Q*_*p*_]/*F*_obs_]} (shown in Figure 4) according to (4) and the results are listed in Table 2. It can be inferred from Table 2 that the number of binding sites *n* was approximately equal to 1, indicating that there was one binding site in HAase for liquiritigenin during their interaction. The value of *K*_*a*_ was of the order of 10^4^ L mol^−1^, which indicated that a strong interaction existed between liquiritigenin and HAase.

### 3.3. Binding Mode

The interaction forces between a small molecule and macromolecule include hydrophobic interactions, electrostatic forces, van der Waals forces, and hydrogen bonds [19]. The thermodynamic parameters of binding reaction are the main evidence for confirming the binding force. If there is no significant change of temperature, enthalpy change (Δ*H*°) can be regarded as a constant, and then the values of Δ*H*°, entropy change (Δ*S*°), and free energy change (Δ*G*°) can be obtained from the following equations:

(5) log⁡Ka=−ΔH°2.303RT+ΔS°2.303R,ΔG°=ΔH°−T·ΔS°,

where *R* is the gas constant and *T* is absolute temperature.

According to (5), the values of Δ*H*°, Δ*S*°, and Δ*G*° for the liquiritigenin-HAase interaction are presented in Table 2. The Δ*G*° at 293 and 310 K are both negative, indicating that the binding process is spontaneous. The negative Δ*H*° and positive Δ*S*° mean that electrostatic interaction plays a major role in the formation of the liquiritigenin-HAase complex. However, the value of Δ*H*° is close to zero, indicating that hydrophobic interactions also play a very important role in the formation of the complex. Therefore, the interaction force in the binding process is mainly electrostatic and hydrophobic interactions.

### 3.4. Energy Transfer

**Figure 5:**
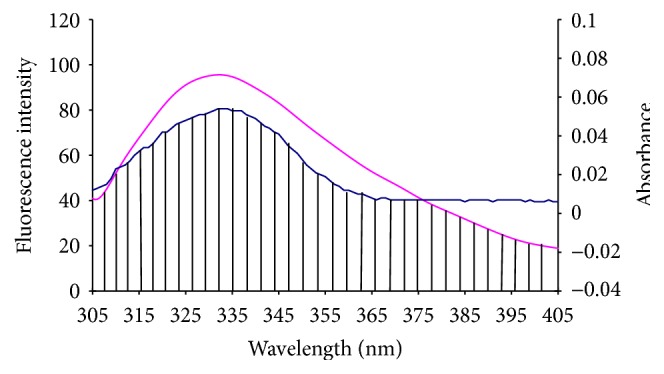
Overlapping of fluorescence spectra of HAase (*C*_HAase_ = 2.0 × 10^−6^ mol L^−1^) with absorption spectra of liquiritigenin (*C*_liquiritigenin_ = 2.0 × 10^−6^ mol L^−1^).

According to Föster's nonradioactive energy transfer theory [20], if an acceptor could absorb the emitted fluorescence from a donor, energy may transfer from the donor to the acceptor. The distances (*r*) between donor (HAase) and acceptor (liquiritigenin) could be determined. The efficiency of energy transfer (*E*) related to the distance:

(6) $$\begin{array}{c} E=1-\frac{F_{\text{obs}}}{F_{\text{cor}}}=\frac{{R_{0}^{}}^{6}}{\left({R_{0}^{}}^{6}+r^{6}\right)}, \end{array}$$

where *R*_0_ is the critical distance when their transfer efficiency is 50%. It is given by the following equation:

(7) $$\begin{array}{c} {R_{0}^{}}^{6}=8.8\times 10^{-25}K^{2}\cdot \Phi \cdot n^{-4}\cdot J, \end{array}$$

where *K* ^2^ is the spatial orientation factor of dipole for random orientations as in a fluid solution, *n* is the refractive index of medium, Φ is the fluorescence quantum yield of donor in the absence of acceptor, and *J* is the overlap integral between the donor fluorescence emission spectrum and the acceptor absorption spectrum (shown in Figure 5), which could be calculated by the equation:

(8) $$\begin{array}{c} J=\frac{\sum \left(F\left(\lambda \right)\cdot \varepsilon \left(\lambda \right)\cdot \lambda^{4}\cdot \Delta \lambda \right)}{\sum \left(F\left(\lambda \right)\cdot \Delta \lambda \right)}, \end{array}$$

where *F*(*λ*) is the fluorescence intensity of donor in the wavelength range *λ* to *λ* + Δ*λ* and *ε*(*λ*) is the molar absorption coefficient of acceptor at wavelength *λ*.

In the present case, *K* ^2^ = 2/3, *n* = 1.366, and Φ = 0.118 [21]. According to (6)–(8), the values of the parameters were calculated to be *J* = 1.899 × 10^−14^ cm^3^ moL^−1^ L, *E* = 0.07, *R*_0_ = 3.23 nm, and *r* = 5.04 nm. The distance between liquiritigenin and HAase was obviously less than 7 nm and 0.5*R*_0_ < *r* < 2.0*R*_0_, indicating that the energy transferring from HAase to liquiritigenin occurs with high possibility.

### 3.5. Conformational Investigations

In order to understand the possible effect of liquiritigenin binding on the secondary structure of HAase, the synchronous fluorescence and the three-dimensional fluorescence spectra were measured in the absence and presence of liquiritigenin.

**Figure 6:**
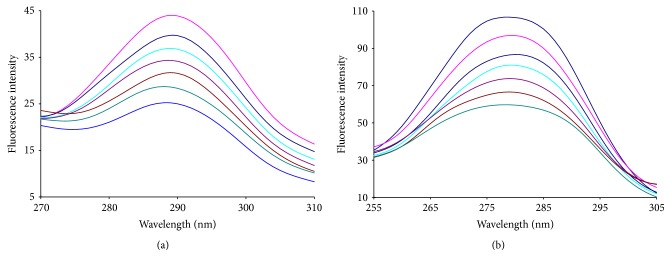
Synchronous fluorescence spectra of interaction between HAase and liquiritigenin at (a) Δ*λ* = 15 nm and (b) Δ*λ* = 60 nm at room temperature. Peak from up to down *C*_liquiritigenin_ = (0, 4.0, 8.0, 12.0, 16.0,20.0, 24.0, 30.0) × 10^−6^ mol L^−1^, *C*_HAase_ = 2.0 × 10^−6^ mol L^−1^.

**Figure 7:**
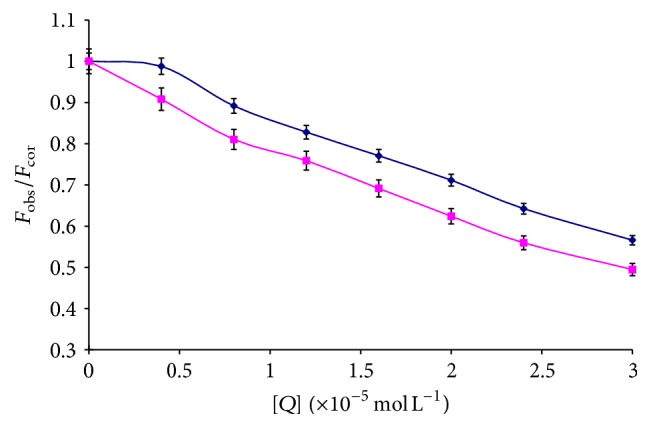
The quenching of HAase synchronous fluorescence by liquiritigenin. *C*_HAase_ = 2.0 × 10^−6^ mol L^−1^, (*◆*) Δ*λ* = 15 nm and (■) Δ*λ* = 60 nm (*n* = 3).

When the wavelength interval (Δ*λ*) between the excitation and emission wavelength is stabilized at 15 or 60 nm, the synchronous fluorescence gives characteristic information of tyrosine (Tyr) residues or tryptophan (Trp) residues, respectively [22, 23]. The synchronous fluorescence spectra at these two different wavelength intervals are presented in Figure 6. As the concentration of liquiritigenin increased gradually, the synchronous fluorescence intensity decreased; however, an obvious shift of the Tyr peak and Trp peak cannot be observed in Figures 6(a) and 6(b), which indicated that liquiritigenin has a weak effect on the microenvironment of Tyr and Trp residues in HAase. On the other hand, it can be seen from Figure 7 that the slope was higher when Δ*λ* was 60 nm, which indicated that liquiritigenin was closer to the Trp residues than to the Tyr residues and the microenvironments of Trp residues were influenced more than those of Tyr residues.

**Figure 8:**
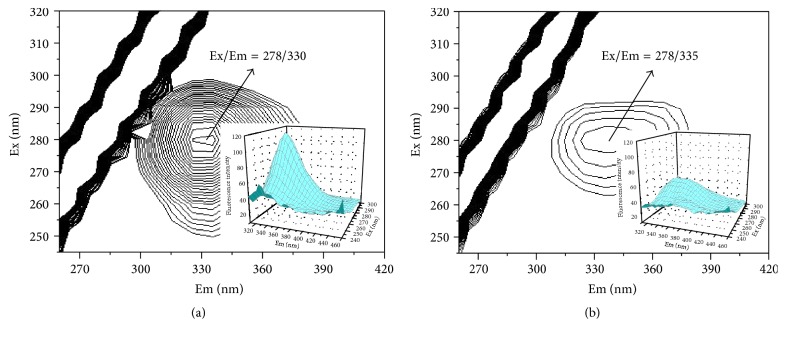
The three-dimensional fluorescence contour spectra of (a) HAase and (b) liquiritigenin-HAase system. (a) *C*_HAase_ = 2.0 × 10^−6^ mol L^−1^, *C*_liquiritigenin_ = 0.0 mol L^−1^; (b) *C*_HAase_ = 2.0 × 10^−6^ mol L^−1^, *C*_liquiritigenin_ = 3.0 × 10^−5^ mol L^−1^.

Three-dimensional fluorescence spectroscopy is a powerful method for providing conformational and structural information of proteins [24]. The three-dimensional fluorescence spectra of HAase and liquiritigenin-HAase systems are shown in Figure 8. It can be seen from Figure 8(a) that the three-dimensional fluorescence contour spectrum of HAase shows contour maxims at *λ*_ex_/*λ*_em_ = 278/330 nm arising by *π*-*π* ^*∗*^ transition of aromatic amino acids in HAase. In Figure 8(b), the HAase fluorescence peak shifted to 278/335 nm. The stoke shift of HAase (*λ*_em_ − *λ*_ex_ = 52 nm) and liquiritigenin-HAase (*λ*_em_ − *λ*_ex_ = 57 nm) was different obviously. The above phenomena and the analysis of the fluorescence characteristic of the peaks revealed that the binding of liquiritigenin to HAase induced some microenvironmental and conformational changes in HAase and a complex between liquiritigenin and HAase has formed. On the other hand, the relative fluorescence intensity of HAase peak was 115.10 in the absence of liquiritigenin. After the addition of liquiritigenin, the relative fluorescence intensity of peak decreased to 50.68. The relative fluorescence intensity of HAase peak decreased a lot after the addition of liquiritigenin, which implied that the peptide chain structure of HAase was changed and this result was consistent with that of synchronous fluorescence spectra.

### 3.6. Modeling Study of the Interaction between Liquiritigenin and HAase

**Figure 9:**
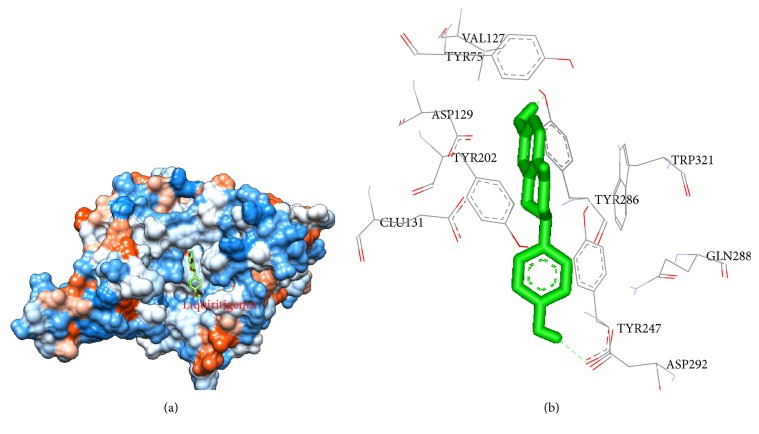
Docked pose corresponding to the minimum energy conformation for liquiritigenin binding to HAase. (a) The hydrophobicity of HAase with liquiritigenin. (b) Detailed illustration of the amino acid residues lining the binding site in the HAase cavity. Green molecule displays liquiritigenin; broken lines display hydrogen bonds.

In order to identify the precise binding sites on HAase, a docking program was performed to simulate the binding mode between HAase and liquiritigenin. The docking results were shown in Table 3. It can be seen from Table 3 that 2 conformations for liquiritigenin were achieved and the observed free energy changes of binding for liquiritigenin-HAase complex were found to be −6.36 kcal mol^−1^ (−26.60 kJ mol^−1^) and −6.24 kcal mol^−1^ (−26.10 kJ mol^−1^), respectively, which was extremely close to the experimental data (−25.7851 and −27.2511 kJ mol^−1^). However, the exact binding sites of liquiritigenin on HAase in these two conformations are different. The exact binding site of liquiritigenin on HAase with the lowest binding free energy is presented in Figure 9, which revealed the most likely binding site in the enzyme. As shown in Figure 9, liquiritigenin was located in the HAase cavity and surrounded by 10 amino acid residues, including 6 hydrophobic amino acid residues (Tyr75, Val127, Tyr202, Tyr247, Tyr286, and Trp321) and 4 hydrophilic amino acid residues (Asp129, Glu131, Gln288, and Asp292). Therefore, it can be concluded that the interaction between liquiritigenin and HAase was mainly electrostatic and hydrophobic forces in nature. The result was in accordance with that of thermodynamic parameter analysis. Furthermore, there also existed two hydrogen bonds between liquiritigenin and Tyr286 (H-band distance, 2.0958) and Asp292 (H-band distance, 2.0797) residues of HAase.

In addition, as shown in Figure 9(b), liquiritigenin also bound with two aspartate residues (Asp129 and Asp292). It was reported that the catalytic site of some enzyme (such as pepsin [25] and rennin [26]) was formed by these two amino acid residues for the protein to be active. Therefore, according to docking and fluorescence results, it could be speculated that liquiritigenin bound directly into the enzyme cavity site and the binding of liquiritigenin into the enzyme cavity influenced the microenvironment of the catalytic site, which would affect the activity of HAase.

## 4. Conclusion

In this study, the binding of liquiritigenin with HAase has been investigated by multispectroscopic and molecular docking methods. Liquiritigenin effectively quenched the fluorescence of HAase by a static quenching process. Based on the results of binding capacity, thermodynamic parameters, and molecular docking study, it was concluded that the liquiritigenin could spontaneously bind with HAase mainly through electrostatic and hydrophobic forces. The synchronous and three-dimensional fluorescence spectra revealed that the microenvironment and conformation of HAase were changed in the presence of liquiritigenin. Since the binding of liquiritigenin affected the microenvironment of the HAase activity site, liquiritigenin caused the inhibition of HAase activity.

## Figures and Tables

**Table 1:** Stern-Volmer constants for the interaction of HAase with liquiritigenin at different temperatures (*n* = 3).

*T* (K)	Equations	*K*_sv_ (L mol^−1^)	*K*_*q*_ (L mol^−1^)	*R* ^a^	SD^b^
293	*F*_obs_/*F*_cor_ = 4.4157[*Q*] + 0.8483	4.4157 × 10^4^	4.4157 × 10^12^	0.9937	0.15
310	*F*_obs_/*F*_cor_ = 3.9668[*Q*] + 0.9838	3.9668 × 10^4^	3.9668 × 10^12^	0.9942	0.11

^a^The correlation coefficient.

^b^The standard deviation.

**Table 2:** The binding constant *K*_*a*_ and relative thermodynamic parameters of the liquiritigenin-HAase system (*n* = 3).

*T* (K)	*K*_*a*_ (L mol^−1^)	*n*	*R* ^a^	SD^b^	Δ*H*° (kJ mol^−1^)	Δ*G*° (kJ mol^−1^)	Δ*S*° (J mol^−1^ K^−1^)
293	3.9537 × 10^4^	1.0623	0.9928	0.09	−0.5174	−25.7851	88.9001
310	3.9079 × 10^4^	1.4084	0.9988	0.07	−0.5174	−27.2511	87.9052

^a^The correlation coefficient.

^b^The standard deviation.

**Table 3:** The lowest energy-ranked results of two liquiritigenin-HAase binding conformations.

Energy-ranked results	Conformation data	
	1	2
Binding energy (kcal mol^−1^)	−6.36	−6.24
Ligand efficiency (kcal mol^−1^)	−0.33	−0.33
Inhibition constant (*μ*M)	21.75	26.53
Intermolecular energy (kcal mol^−1^)	−6.91	−6.79
